# Indirect reciprocity undermines indirect reciprocity destabilizing large-scale cooperation

**DOI:** 10.1073/pnas.2322072121

**Published:** 2024-04-29

**Authors:** Eric Schnell, Michael Muthukrishna

**Affiliations:** ^a^Department of Psychological and Behavioural Science, London School of Economics and Political Science, London WC2A 2AE, United Kingdom

**Keywords:** cooperation, indirect reciprocity, cultural evolution, evolutionary game theory

## Abstract

Previous models suggest that indirect reciprocity (reputation) can stabilize large-scale human cooperation [K. Panchanathan, R. Boyd, *Nature* **432**, 499–502 (2004)]. The logic behind these models and experiments [J. Gross *et al.*, *Sci. Adv.* **9**, eadd8289 (2023) and O. P. Hauser, A. Hendriks, D. G. Rand, M. A. Nowak, *Sci. Rep.* **6**, 36079 (2016)] is that a strategy in which individuals conditionally aid others based on their reputation for engaging in costly cooperative behavior serves as a punishment that incentivizes large-scale cooperation without the second-order free-rider problem. However, these models and experiments fail to account for individuals belonging to multiple groups with reputations that can be in conflict. Here, we extend these models such that individuals belong to a smaller, “local” group embedded within a larger, “global” group. This introduces competing strategies for conditionally aiding others based on their cooperative behavior in the local or global group. Our analyses reveal that the reputation for cooperation in the smaller local group can undermine cooperation in the larger global group, even when the theoretical maximum payoffs are higher in the larger global group. This model reveals that indirect reciprocity alone is insufficient for stabilizing large-scale human cooperation because cooperation at one scale can be considered defection at another. These results deepen the puzzle of large-scale human cooperation.

## Significance

The emergence of large-scale cooperation remains one of the great scientific puzzles across many disciplines. Previous models have suggested that indirect reciprocity is sufficient to sustain large-scale cooperation, but these models assume that people only belong to one cooperative group. In reality, people belong to multiple cooperative groups with different, often competing incentives. Here, we extend these models of indirect reciprocity showing that under a range of realistic conditions, reputation at a lower scale of cooperation (smaller group) will undermine reputation at a higher scale of cooperation (larger group).

Human cooperation takes many forms, occurring at different scales in different societies, multiple scales within the same society, and across different domains (1). In some societies, people primarily cooperate with extended families (2, 3) and in others, cooperation occurs across large nation-states and diverse ethnic populations (4, 5). The coexistence of scales can create problems such as corruption and nepotism, which can be interpreted as small-scale cooperation with family and friends undermining large-scale cooperation with society as a whole (6, 7). Indirect reciprocity has been proposed as a mechanism for aligning incentives to cooperate across multiple scales.

In a seminal model, Panchanathan and Boyd (8) model a two-step cooperative game, where players play a Public Goods Game (PGG) followed by a Mutual Aid Game (MAG). In the PGG, players can apportion some of their endowment to a public good which is multiplied and then divided evenly among all players regardless of contribution. The multiplier (M) is less than the number of players (N; M<N), such that the Nash equilibrium is to contribute nothing to the public good. Players are then paired with group members at random and are given the choice to provide them with aid, which costs the provider and only benefits the receiver. The model reveals that an evolutionary stable strategy is for players to conditionally aid those who cooperate in the PGG and not those who defect. In this way, the MAG serves as a way to reward cooperators and punish defectors. Thus, indirect reciprocity—reputation for cooperation in the PGG—can maintain large-scale cooperation without the second-order free-rider problem. Later experiments support the insights from this model (9).

Panchanathan and Boyd’s (8) model and other similar models of indirect reciprocity (10–14) fail to account for the existence of multiple possible cooperative reputations because people belong to multiple groups—multiple PGGs. For example, one could donate money to a local conservation group maintaining local parks or to a national or even international conservation group. In either case, this individual is donating to a cause that benefits others, but the scale of the cause and the circle of affected individuals differs. As an outside observer, how do we weigh these different actions and which do we choose to prioritize for improving a person’s reputation (15, 16)? If cooperation at the local level is continuously prioritized over cooperation at the global level, this risks eroding large scale cooperation. This can be thought of as corruption, where small subsets of a larger group cooperate together at the detriment of the larger group. Existing models of indirect reciprocity fail to consider the implications of how different scales of cooperation interact on whether these scales can be aligned.

Here, we extend Panchanathan and Boyd’s (8) model such that players belong to multiple groups. In our model, players belong to one of a groups, which we call local groups. These groups and all players also belong to a larger cooperative group, which we call the global group. Local groups are all the same size and independent of one another. Players play two simultaneous PGGs where they can contribute to the local PGG, the global PGG, or defect. Players then play a MAG with a randomly chosen member of their local group. In keeping with previous work on indirect reciprocity, positive reputation in our model is determined by the leading eight strategies of indirect reciprocity—eight social norm strategies that can sustain cooperation through reputation-based indirect reciprocity (15, 17). We analyze our model for all 8 strategies and although different strategies do have effects on specific invasion dynamics, they don’t change the general pattern of results. As such, throughout the paper, we will only be discussing the results for using one of these strategies, the standing strategy, where cooperating always leads to a positive reputation and defecting from aiding a player of positive reputation makes you lose your positive reputation. We use this strategy to be directly comparable to Panchanathan and Boyd’s (8) model, allowing us to replicate and then show the limitations of their results. Analysis using the other leading eight strategies can be found in *SI Appendix*. We analyze evolutionarily stable strategies using an adaptive dynamics approach, testing invasibility of 15 possible strategies against each other for a single rare mutant of each strategy and a group of individuals with each strategy. These 15 strategies are composed of three possible PGG strategies and five possible MAG strategies.

**Fig. 1.**
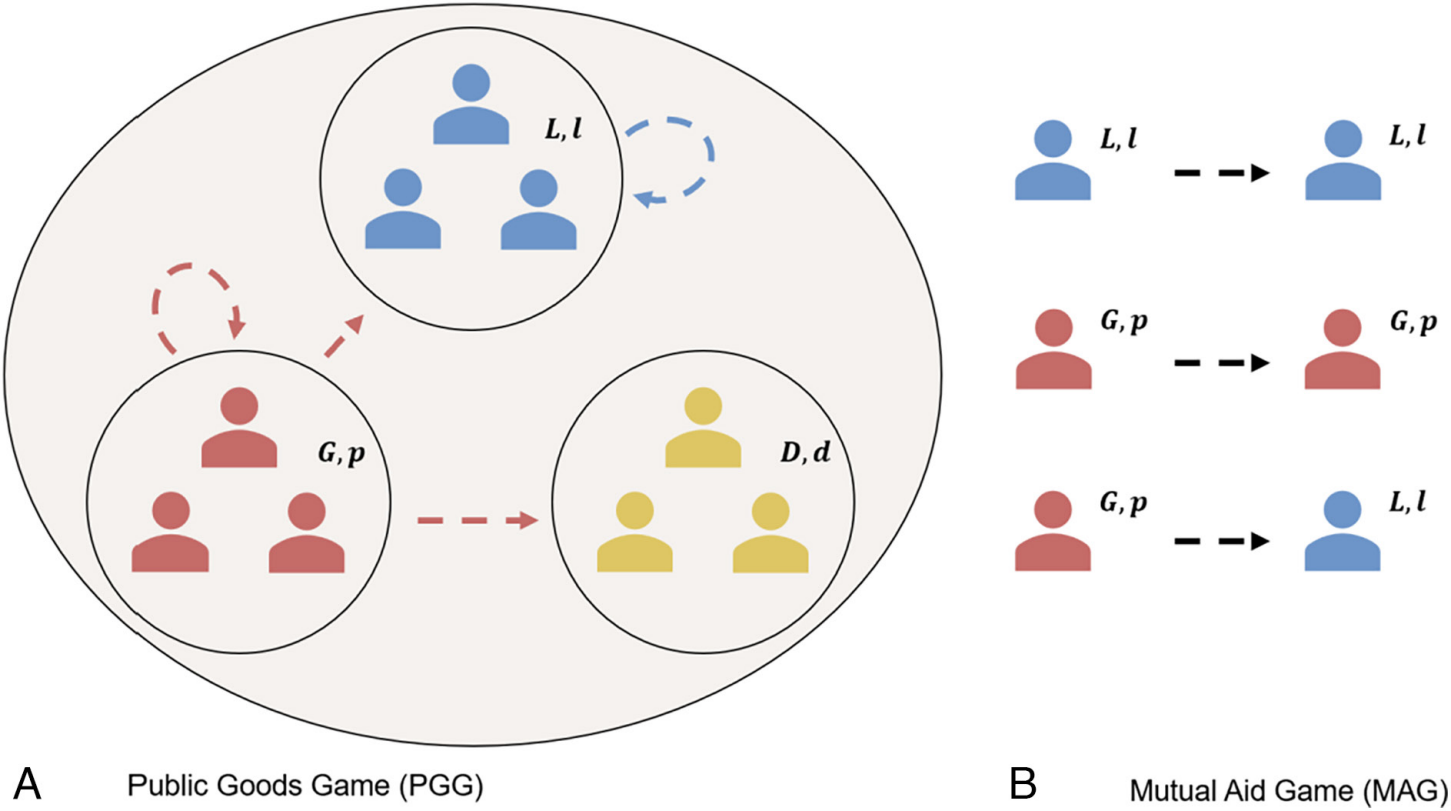
(*A*) Shows a perspective payout from different PGG strategies. In this example, players using strategies (G,p), (L,l) and (D,d) are each found in their own group with arrows showing how PGG returns are distributed. (*B*) Shows how prospective pairings provide aid in the MAG. In this example, players using (G,p) help those who provided to either PGG and so aid players using (L,l), however (L,l) only provides aid to local cooperators and so won’t aid (G,p) in return. Crucially, this presents a second-order free-rider problem, someone who cooperates in the first instance but won’t provide aid to cooperators in the second instance, showing the need for MAG strategies such as pm.

For the PGG players can either always cooperate in the global PGG (G), always cooperate in the local PGG (L), or defect, cooperating in neither (D). We also modeled intermediate strategies contributing 25%, 50%, or 75% to each of the local and global PGGs, but as we discuss in *SI Appendix*, strategies contributing to either local or global always dominate over these intermediate strategies. For the MAG, players can either always provide aid (c), provide aid only to global PGG cooperators (g), provide aid only to local PGG cooperators (l), provide aid to others who aid in the MAG (m), provide aid to those who cooperated in the PGG and provided aid in the MAG (pm), or never provide aid to anyone (d). An example of how some of these strategies interact is shown in Fig. 1.

For completeness, in *SI* we also analyze PGG strategies which are dependent on having received aid in the MAG. These strategies do withstand invasion from defectors better than their more cooperative counterparts, but in turn they are less able to invade other strategies and they are prone to being invaded by PGG strategies without these dependencies. For simplicity, we will omit discussing these throughout the rest of the paper as the main results of our model are best illustrated by their more cooperative counterparts.

Overall, we find that consistent with previous models of large-scale cooperation sustained by indirect reciprocity, when there is effectively only one PGG and MAG [i.e., strategies (G,pm) and (L,pm)], indirect reciprocity is sufficient to sustain cooperation. That is, defectors [i.e., (G,d), (L,d), and (D,d)] cannot invade these strategies. However, when there are multiple scales of cooperation, then the smaller scale is more stable even when the multiplier and potential payoffs are higher in the global PGG, because fewer people need to cooperate in the local PGG. Conflicting reputations lead to smaller-scale, local cooperation undermining larger-scale, global cooperation.

## Results

**Fig. 2.**
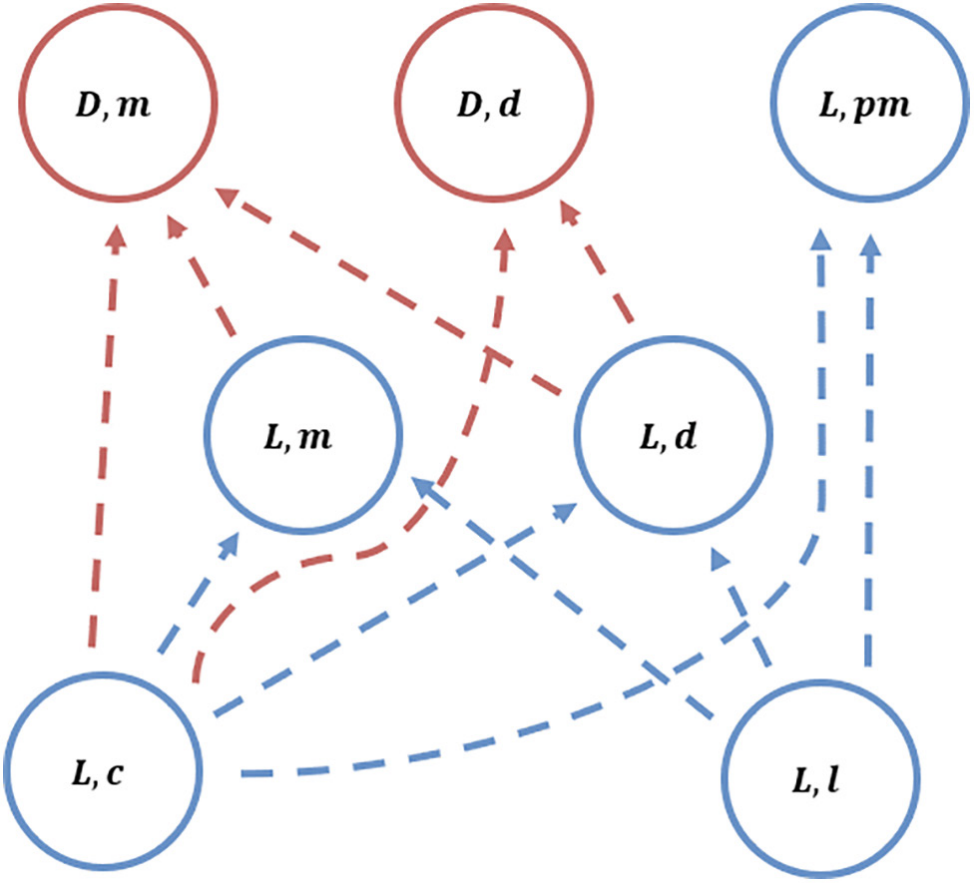
Example of invasion pathway for a single invader for parameters ($c_{p}=1;\;c_{m}=1;\;b_{m}=3;\;b_{g}=3;\;b_{l}=2;\;n_{l}=5;\;a=5;\;e=0.05;\;\beta =0$). Given the resident using one of the circled strategies, arrows to other strategies show successful invasions by mutants. As a result, strategies at the *Bottom* of the diagram will always be invaded and are unable to invade others, strategies at the *Top* of the diagram invade others and are stable and strategies in the middle of the diagram invade some strategies but get invaded by others. For clarity, we’ve highlighted only the local scale of cooperation (in blue) and PGG defectors (in red), but the global scale behaves the same.

We first analyze whether a single rare mutant of each strategy can invade a resident population of each other strategy. Consistent with Panchanathan and Boyd (8), when there’s only one rare invader, defectors—PGG (D) and either defect (d) or reciprocate (m) in the MAG—can invade all other strategies, except for cooperative strategies utilizing indirect reciprocity, such as (G,g) or (G,pm). Such cooperative strategies utilizing indirect reciprocity are resistant to direct invasion by defectors but can’t invade these defectors in return. Furthermore, a reciprocal strategy that only cares about one of the PGG and MAG, such as (G,g) or (G,m), can be invaded by defectors in the long run-through intermediate strategies which continue to cooperate in the PGG. This invasion pathway is shown in Fig. 2. To prevent invasion of defector strategies, a resident population of PGG cooperators must have an MAG strategy that discriminates both on previous PGG and MAG behavior when deciding who to aid, such as (G,pm) or (L,pm). That is, strategies in which players fall out of good standing if they’ve defected from either the PGG or MAG. These cooperative strategies while resistant to invasion from rare defectors also can’t invade a resident population of defectors. Thus, indirect reciprocity can ensure cooperators resist invasion from defectors, but a rare mutation of cooperation is not a solution how cooperation could evolve in the first place (18).

Cooperative benefits only emerge when there is more than one cooperator. This means, for cooperative strategies to invade others, mutants must invade in groups. As such, we next consider some percentage β of players in one local group with an invading strategy, with the rest of the resident local group (1-β) as well as all other local groups using a resident strategy. We start by analyzing a specific case of this approach where β=1, or the entirety of one local group uses the invading strategy. Our analyses reveal that defecting strategies can only invade global cooperator strategies, not local cooperator strategies, and can only do so when

[1] $$\frac{b_{g}}{a}+\left(1-e\right)\cdot \left(b_{m}-c_{m}\right)<c_{p}.$$

**Fig. 3.**
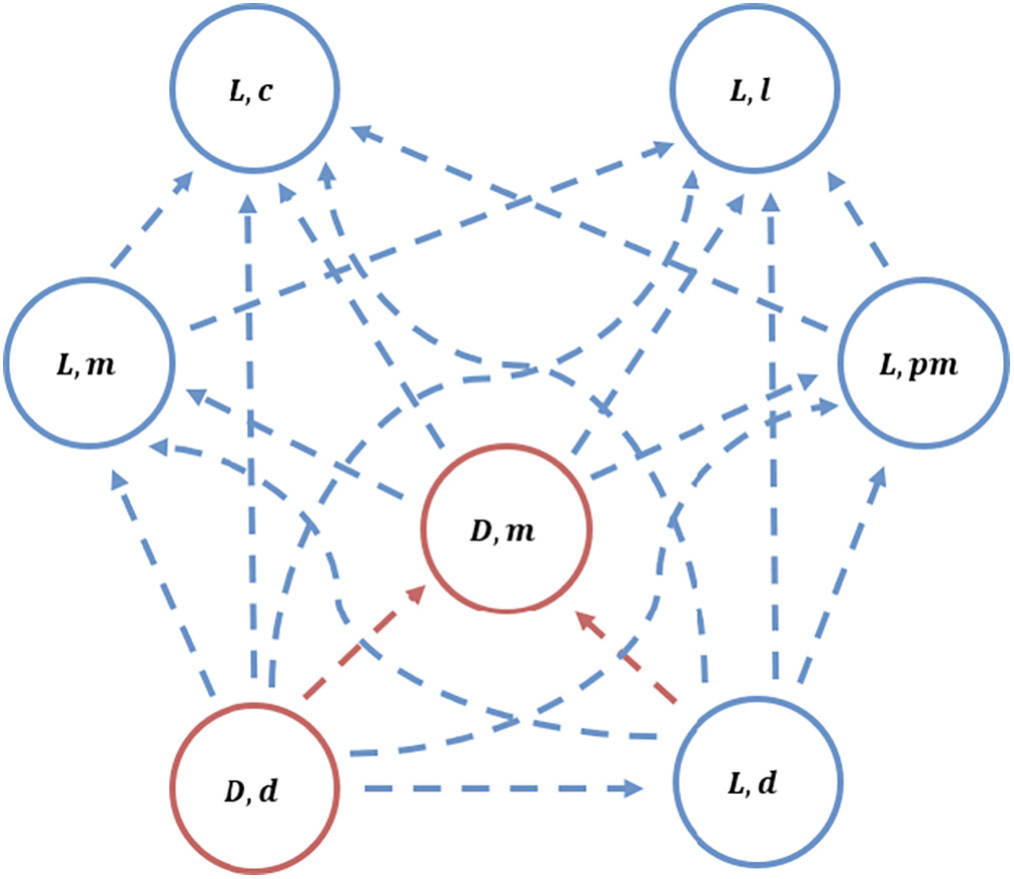
Example of invasion pathway for a group of invaders for parameters ($c_{p}=1;\;c_{m}=1;\;b_{m}=3;\;b_{g}=3;\;b_{l}=2;\;n_{l}=5;\;a=5;\;e=0.05;\;\beta =1$). Given the resident using one of the circled strategies, arrows to other strategies show successful invasions by mutants. As a result, strategies at the *Bottom* of the diagram will always be invaded and are unable to invade others, strategies at the *Top* of the diagram invade others and are stable and strategies in the *Middle* of the diagram invade some strategies but get invaded by others. For clarity we’ve highlighted only the local scale of cooperation (in blue) and PGG defectors (in red), but the global scale behaves the same.

In other words, defectors can invade if the cost of cooperating in the PGG outweighs both the PGG benefits and net benefits from aiding and being aided in the MAG. This invasion pathway is shown in Fig. 3. The reason defectors can invade at all is that they continue to free-ride on the benefits provided by global cooperators in other local groups. For this same reason, defectors can’t invade local cooperators, because they don’t receive any benefits from the other local groups.

When comparing how each scale of cooperation interacts, local cooperators will invade global cooperators as long as

[2] $$b_{l}<\frac{b_{g}}{a}.$$

That is, for global cooperation to outcompete local cooperation requires benefits to be higher than the benefits of all local groups combined (i.e., $a\cdot b_{l}$). Unless global cooperators can support all other local groups cooperating at a different scale, then local cooperation will undermine global cooperation even when the benefits of cooperating at a global scale are higher. Moreover, the more splintered the society is—the more local groups there are—the harder it is for global cooperation to outcompete local cooperation. We expand upon this insight by analyzing how varying the size of one of the local groups effects this relationship. As the local group approaches the size of the global group, global cooperation becomes more competitive. Intuitively, this is because global cooperators are able to police a larger proportion of the population. The reason for local cooperation’s emergence to begin with, is the result of players having the ability to directly condition the behavior of other local cooperators. This makes local cooperation much more stable and likely to emerge than global cooperation under a range of realistic parameters.

When β<1, then the number of invaders is variable. A β value closer to 1 will resemble the group invasion results and β closer to 0 will resemble the single invader results. That is to say, we expect cooperation to be more stable for higher β values, which is exactly what we see in Eqs. **3** and **4**. Eq. **3** shows the criteria for defectors to invade and dominate global cooperators:

[3] $$\frac{b_{g}\cdot \beta }{a}+\left(1-e\right)\cdot (b_{m}-c_{m})<c_{p}.$$

In addition, Eq. **4** shows when defectors invade local cooperators:

[4] $$b_{l}\cdot \beta +\left(1-e\right)\cdot (b_{m}-c_{m})<c_{p}.$$

The inequalities are similar to the group of invaders condition, but the benefits of the PGG are lessened because there are fewer players contributing to it. Regardless of the direction of invasion, when players split their actions between cooperating and defecting, then invasion becomes likely. Eqs. **3** and **4** show that cooperators can only avoid a free-ridership problem when there are either very few defectors or the rewards for cooperation, in both the PGG and MAG, are sufficiently high to outweigh the potential benefits of defecting. Comparing local and global cooperators, there exists a similar issue of splitting strategies. This is not quite the same as a free-rider problem as all players cooperate, but we similarly find that the rate of invaders will determine whether an invasion is successful. In Eq. **5**, we show the conditions for local cooperators to invade global cooperators:

[5] $$\frac{b_{g}\cdot \beta }{a}+\left(1-\beta \right)\cdot \left(1-e\right)\cdot \left(b_{m}-c_{m}\right)<b_{l}\cdot \beta .$$

**Fig. 4.**
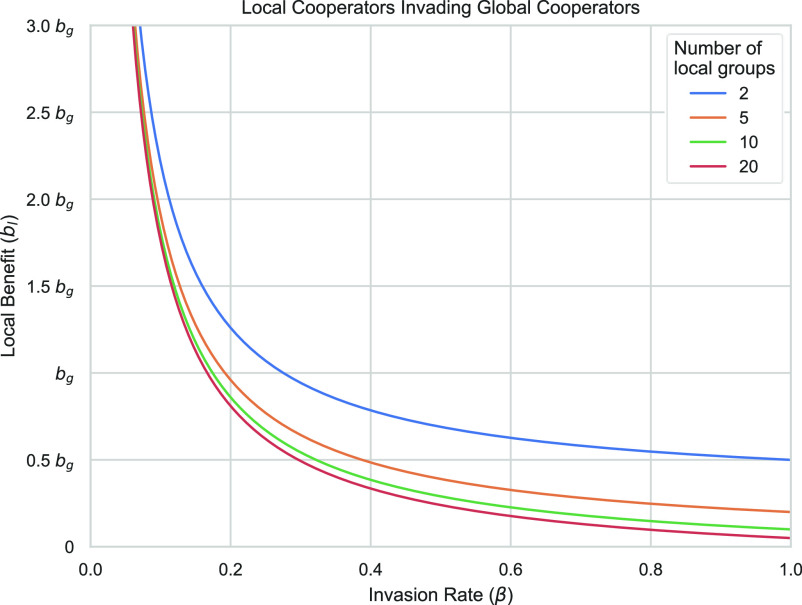
Minimum benefit of local cooperation ($b_{l}$), in relation to the benefit of global cooperation ($b_{g}$), required for local cooperators to invade global cooperators, given an invasion rate (β). Values of $b_{l}$ greater or equal to those listed will ensure that local cooperators successfully invade global cooperators. Note that for $b_{l}<b_{g}$ global cooperation provides a greater max return (all players globally cooperating is better than all players locally cooperating) and yet local cooperators can still invade global cooperators. As the number of mutants using a shared strategy increases (β), the required benefits of local cooperation needed for invasion decreases. When the global group is more fractured, i.e., there are more local groups, then the required benefit for local cooperators to invade is lower. Parameters shown: $b_{g}=5$, $c_{m}=1$, $b_{m}=2$, e=0.05.

Here, cooperators determine the size of the payouts for each PGG as well as whether players will be aided in the MAG. Note that there are MAG strategies such as m, p, and pm which may aid both local and global cooperators in this case. As Fig. 4 reveals, as the rate of local cooperator invaders (β) increases, the required returns from local cooperation ($b_{l}$) in relation to global cooperation ($b_{g}$) for there to be a successful invasion decreases. Furthermore, when the global group is fractured into more local groups (a is bigger), then local cooperation emerges under even lower payouts and is thus considerably more stable than global cooperation.

Thus, under a wide range of conditions and arguably all realistic conditions, indirect reciprocity when there is more than one possible reputation leads to lower scales of cooperation undermining higher scales. Global cooperation can only dominate when the global PGG benefit is so high that there is effectively no longer a dilemma between contributing to the local and global group. Thus, indirect reciprocity alone is not sufficient to sustain large-scale human cooperation.

## Discussion

Individuals rely on the reputation of others when making cooperative decisions (19–25). However, often these reputations can be in conflict (11, 26, 27). The same person may have a positive reputation with some groups and a negative reputation with others in the same society; the same person may have a positive reputation in some domains and a negative reputation in others. This makes it difficult to determine with whom you should cooperate. Given that societies are made up of overlapping and embedded groups of differing sizes and scales of cooperation, it is necessary to reconcile reputational differences across different scales of cooperation.

This model shows how different scales of cooperation interact revealing that small-scale cooperation in a local group is more likely to be sustained than large-scale cooperation, even when cooperation is more beneficial at the larger scale. These results add further insight to an emerging literature on intergroup interactions. If group members interact more frequently across local group boundaries and move between groups, the effective population becomes closer to the global population incentivizing higher-scale cooperation (10, 28). By corollary, if individuals are more likely to interact within a local group (e.g., local region or ethnic boundary), then local cooperation will dominate. Similarly, this model is consistent with corruption as a lower-scale of cooperation undermining a higher scale (1, 6, 7) and supports findings that lower-scale cooperation is more stable than a higher scale (23, 29, 30). If an invasion by local cooperators is possible in the first place, then inevitably all other local groups will also convert to cooperating locally. This suggests that small-scale corruption erodes cooperation at higher scales and can lead to fracturing within a society. Because corruption as lower-scale cooperation within a large group will degrade the possibility of all group members working together, any society with some corruption risks descending into a wholly corrupt system where cooperation only occurs within local groups. Finally, the model is also related to research on parochial cooperation. Research shows that in-group favoritism leads to a preference for working with like-minded local group rather than a diverse global group (31–35). These psychological mechanisms may be a proximate manifestation of the ultimate dynamics of overlapping scales of cooperation modeled here (36, 37).

Our model explores how multiple overlapping cooperative groups compete with one another. However, we don’t consider the multitude of ways in which cooperation can be further complicated, such as through noisy reputational information (11, 25) or by varying the way in which a person’s reputation is decided (35, 38, 39). As such, although this model relies on reputation being made messy by group dynamics, there are other ways to also make cooperation based on reputation messy. We hope to further expand upon this work by further analyzing how reputation is instantiated in practice.

Indirect reciprocity maintains cooperation at different cooperative scales. These multiple scales of indirect reciprocity protect cooperators from descending into full defection, but they also compete with and undermine one another. As such, under a range of conditions, societies are at risk of collapsing to lower scales of cooperation, which may help explain fracturing and corruption in previously cooperative societies as a result of resource constraints or slowed economic growth reducing payoffs at a larger societal scale.

## Materials and Methods

We consider a group of $n_{g}$ individuals, which we will call our global group, subdivided into a groups of $n_{l}$ individuals, which we will call local groups. Each member of the global group is assigned to one local group and each local group is independent from one another, which implies $n_{g}=a\cdot n_{l}$. Players begin by playing a PGG with two investment pools, a global pool, which everyone has access to, and a local pool, which is unique to each local group and only that local group has access to. To cooperate in the PGG costs the cooperator $c_{p}$ which becomes $b_{g}$ if invested in the global pool or $b_{l}$ if invested into the local pool. The relationship between $b_{g}$ and $b_{l}$ is undetermined, with it being possible for either game to be more beneficial. The PGG returns are then divided evenly among the respective groups. Players then play a MAG within their local group. Here, they are paired with one random member of their local group and with each partner they can choose to pay a cost $c_{m}$ which yields their partner a benefit of $b_{m}$. In turn, each player is also the receiving partner of another local group member, where they receive $b_{m}$ when their partner pays the cost associated. Players know each other’s action from the previous round of the PGG and MAG, which will be used to determine how players act in future rounds. We also include an implementation error where players will accidentally do the opposite they intended in the MAG. The full list of functions is listed in Table 1 and the full list of parameters is listed in Table 2.

**Table 1.** Model functions

Function	Meaning
F(i,j)	Fitness of player using strategy (i,j)
$F_{p}\left(i,j\right)$	Fitness derived from PGG of player using strategy (i,j)
$F_{m}\left(i,j\right)$	Fitness derived from MAG of player using strategy (i,j)
$V_{g}(i,j)$	Proportion of players using strategy (i,j) who contribute to the global PGG
$V_{l}(i,j)$	Proportion of players using strategy (i,j) who contribute to the local PGG
V(i,j)	Proportion of players using strategy (i,j) who contribute to either PGG. Also defined as $V_{g}(i,j)+V_{l}(i,j)$
H(i,j)	Proportion of local group members who players using strategy (i,j) will provide aid to in the MAG
I(i,j)	Proportion of local group members who provide aid in the MAG to players using strategy (i,j)
$A_{b}(i,j)$	Proportion of bad reputation players who players using strategy (i,j) will aid
$A_{g}(i,j)$	Proportion of good reputation players who players using strategy (i,j) will aid
$D_{b}(i,j)$	Proportion of bad reputation players who players using strategy (i,j) will defect from aiding
$D_{g}(i,j)$	Proportion of good reputation players who players using strategy (i,j) will defect from aiding
X(i,j)	Proportion of good reputation players who players using strategy (i,j) will attempt to aid
Y(i,j)	Proportion of good reputation players who players using strategy (i,j) will attempt to defect from aiding
Z(i,j)	Proportion of players who players using strategy (i,j) will attempt to aid

**Table 2.** Model parameters

Parameter	Meaning	Domain
$y_{i,j}$	Proportion of local group members using strategy (i,j)	$0<y_{i,j}<1$
a	Number of local groups	<0
$n_{l}$	Number of people per local group	<0
$n_{g}$	Number of people in the global group	$=a\cdot n_{l}$
$c_{p}$	Cost of contributing to either PGG	<0
$c_{m}$	Cost of aiding in the MAG	<0
$b_{l}$	Returns from local PGG contributions	$<c_{p}$
$b_{g}$	Returns from global PGG contributions	$<c_{p}$
$b_{m}$	Returns from being aided in the MAG	$<c_{m}$
e	Error rate	0<e<1

The possible strategies for the PGG portion of the game are listed in Table 3 and for the MAG portion these are listed in Table 4. Note that there are more strategies listed here than are discussed in the text’s body. The extra strategies are LG in the PGG, which contributes 25%, 50%, or 75% to each of the local and global PGGs, M and O in the PGG, which contribute to the global and local PGGs, respectively, but only if they’ve been aided in the MAG, and p in the MAG which aids all PGG cooperators regardless of which one that is. The results including LG, M, O, and p are discussed only in *SI Appendix*.

**Table 3.** PGG strategies

PGG strategy	Description
G	Always contributes to the global PGG pool
L	Always contributes to the local PGG pool
LG	Always contributes to both local and global PGG pool
D	Does not contribute to either PGG
M	Contributes to the global PGG pool, only if they received MAG aid
O	Contributes to the local PGG pool, only if they received MAG aid

**Table 4.** MAG strategies

MAG strategy	Description
c	Always provides aid in the MAG
g	Only provides MAG aid to global PGG contributors
l	Only provides MAG aid to local PGG contributors
d	Does not provide MAG aid
p	Provides aid to all PGG contributors, regardless of which pot they contributed to
m	Provides aid to those who aid others in good reputation in the MAG (where players are considered in good reputation if they themselves aid other good reputation players)
pm	Provides aid to those who contributed to either PGG and are in good MAG reputation

[6] $$F\left(i,j\right)=F_{p}\left(i,j\right)+F_{m}\left(i,j\right),$$

[7] $$F_{p}\left(i,j\right)=\frac{\sum_{k}x_{G,k}+\sum_{k}x_{M,k}V_{g}\left(M,k\right)}{\underset{Returns\;from\;the\;global\;PGG}{\underset{︸}{n_{g}}}}b_{g}+\frac{\sum_{k}y_{L,k}+\sum_{k}y_{O,k}V_{l}\left(O,k\right)}{\underset{Returns\;from\;the\;local\;PGG}{\underset{︸}{n_{l}}}}b_{l}-\underset{\begin{array}{c} Cost\;of\;contributing\\ to\;either\;PGG \end{array}}{\underset{︸}{V\left(i,j\right)c_{p}}},$$

[8] $$F_{m}\left(i,j\right)=\frac{\left(1-e\right)\left(n_{l}-1\right)}{n_{l}}\left(\underset{\begin{array}{c} Rate\;of\;group\;members\\ providing\;you\;aid \end{array}}{\underset{︸}{G\left(i,j\right)b_{m}}}-\underset{\begin{array}{c} Rate\;of\;group\;members\\ whom\;you\;aid \end{array}}{\underset{︸}{H\left(i,j\right)c_{m}}}\right).$$

A player’s fitness Eq. **6** is broken down into two parts, PGG fitness Eq. **7** and MAG fitness Eq. **8**. PGG fitness is composed of returns from the global pool, returns from the local pool, and the cost of contributing to either of the pools. MAG fitness is composed of aid received from other local group members minus aid provided to other group members. This portion is determined by yours’ and others’ reputations. In *SI Appendix*, we discuss the leading eight strategies in determining reputation, but here we present a standing strategy of reputation. Players start with a positive reputation and lose their reputation if they fail to aid other positive reputation players. This reputation is only used by players using MAG dependent strategies (m and pm). PGG-dependent strategies such as g and l instead rely on a player’s contributions to the PGG to determine whether to provide MAG aid.

Reputation is operationalized recursively. Players start with a good reputation and each round they can either lose their good reputation, or if it’s already lost then regain it. For a given round n, the percentage of players using strategy (i,j) with a good reputation, with some amount of variation due to a possible error e in implementing their strategy, is given by $W_{n}\left(i,j\right)$:

[9] $$W_{n}\left(i,j\right)=\underset{\begin{array}{c} Previously\;good\;standing\;players\\ who\;maintain\;their\;standing \end{array}}{\underset{︸}{W_{n-1}\left(i,j\right)\left(1-D_{g}(i,j)\right)}}+\underset{\begin{array}{c} Previously\;bad\;standing\;players\\ who\;regain\;good\;standing \end{array}}{\underset{︸}{\left(1-W_{n-1}\left(i,j\right)\right)\left(A_{g}\left(i,j\right)+A_{b}\left(i,j\right)\right)}}.$$

A player’s reputation in round n is dependent on their reputation in the previous round (n-1) and their actions in the previous round (n-1). This formula is itself a product of which leading eight strategy is implemented. Here, we list the formula for the first leading eight strategy, or the standing strategy, but a more general form can be found in *SI Appendix*. In red we have the rate of players using strategy (i,j) who were in good standing previously and remain in good standing based on their actions in the MAG. In blue we have the rate of players using strategy (i,j) who were in bad standing previously but manage to regain a good standing based on their actions in the MAG. At equilibrium this equals:

[10] $$W\left(i,j\right)=\frac{A_{g}\left(i,j\right)+A_{b}\left(i,j\right)}{A_{g}\left(i,j\right)+A_{b}\left(i,j\right)+D_{g}(i,j)}.$$

Finally, the component parts of Eqs. **9** and **10** can themselves be broken down into smaller portions.

[11] $$A_{g}\left(i,j\right)=X\left(i,j\right)\left(1-e\right)+Y\left(i,j\right)e,$$

[12] $$A_{b}\left(i,j\right)=X\left(i,j\right)e+Y\left(i,j\right)\left(1-e\right),$$

[13] $$D_{g}\left(i,j\right)=\left(Z\left(i,j\right)-X\left(i,j\right)\right)e+\left(1-Z\left(i,j\right)-Y\left(i,j\right)\right)\left(1-e\right).$$

Here, X(i,j) is the proportion of players in good MAG standing that players with strategy $\left(i,j\right)$ attempts to give to, Y(i,j) is the proportion of players in good MAG standing that players with strategy $\left(i,j\right)$ attempt to defect from giving to, and Z(i,j) equals all players that players with strategy $\left(i,j\right)$ attempt to give to, both in good and bad MAG standing. These must be defined explicitly for each MAG strategy, but they follow directly from the definition of these strategies. The explicit definitions are described in *SI Appendix*.

## Supplementary Material

Appendix 01 (PDF)

Dataset S01 (XLSX)

Code S01 (TXT)

## Data Availability

All study data are included in the article and/or supporting information.
