# Supplementary material for: Indirect reciprocity undermines indirect reciprocity destabilizing large-scale cooperation
Source: Proc Natl Acad Sci U S A. 2024 Apr 29;121(19):e2322072121. doi: 10.1073/pnas.2322072121 (PMC11087788; doi:10.1073/pnas.2322072121)
Supplement: Supplementary file 1 — Appendix 01 (PDF) [file pnas.2322072121.sapp.pdf]

Supporting Information for

Indirect Reciprocity Undermines Indirect Reciprocity Destabilizing Large-Scale Cooperation

Eric Schnell and Michael Muthukrishna

Corresponding Author Eric Schnell.
E-mail: e.r.schnell@lse.ac.uk

This PDF file includes:

Supporting text
Tables S1 to S4
Legends for Dataset S1 to S2

Other supporting materials for this manuscript include the following:

Datasets S1 to S2

Supporting Information Text

In the supplementary information text we go over how we derive all the functions which allow us to analyze our model. The actual analysis is completed in the python script also provided in the supplementary information. A full table of results can be found in the spreadsheet, also found in the supplementary information.

We begin by writing the payoffs for each strategy. These strategies can be found in tables S1 and S2. There are a total of $6 \times 6 = 36$ strategies. Note strategies LG, M, O and p are omitted from the main text and only considered here. For our purposes we need to define a number of functions, which are listed in table S3. Theses functions are dependent on the parameters of our model, listed in table S4. To begin recall the payoff formulas as stated in the methods of the manuscript:

$$F(i,j) = F_p(i,j) + F_m(i,j) \quad [1]$$

$$F_p(i,j) = \frac{\sum_k x_{G,k} + \sum_k x_{M,k} V_g(M,k) + \sum_k x_{LG,k} V_g(LG,k)}{n_g} b_g + \frac{\sum_k x_{L,k} + \sum_k x_{O,k} V_l(O,k) + \sum_k x_{LG,k} V_g(LG,k)}{n_l} b_l - V(i,j)c_p \quad [2]$$

$$F_m(i,j) = \frac{(1-e)(n_l - 1)}{n_l} \left(I(i,j)b_m - H(i,j)c_m \right) \quad [3]$$

Equations 2 and 3 both rely on defining more functions, specifically related to questions of reputation. Equation 2 relies on defining whether or not someone contributes to a public good (V, V_g, and V_l). Note equation 2 can be written in a more general form where all strategies are checked to see if they give contributions (instead of say only taking V_g for strategies using M in the PGG), but V_g and V_l will be either 1 or 0 for all strategies except M, O and LG, so other strategies can be excluded. Equation 3 relies on defining who a player receives aid from ($I(i,j)$) and who players provide aid to ($H(i,j)$), as determined by reputation.

Note when we run the analysis we assume $\frac{n_l - 1}{n_l} \approx 1$ in equation 3. This makes the analysis easier to interpret without changing the main insights. It should be noted that for sufficiently small local groups (small n_l) this assumption might be unfair. We suspect that if groups are small enough then the returns from the MAG are lessened, potentially dissuading cooperation, but for the purposes of this model we ignore this possibility.

We denote three different types of PGG standing, one in the global PGG (denoted by $V_g(i,j)$), another in the local PGG (denoted by $V_l(i,j)$) and finally an overall PGG standing (denoted by $V(i,j)$), where the overall PGG standing is the sum of these other two numbers ($V(i,j) = V_g(i,j) + V_l(i,j)$). PGG standing is defined for a given round n, denoted by a superscript, and is reliant upon MAG actions, specifically $W(i,j)$ which will be defined shortly. Note that the important variable of the function is the PGG strategy and we will explicitly define the result for each strategy.

$$\begin{aligned}
V_g^n(G,j) &= 1; V_l^n(G,j) = 0 \\
V_g^n(L,j) &= 0; V_l^n(L,j) = 1 \\
V_g^n(LG,j) &= r; V_l^n(LG,j) = 1-r \\
V_g^n(D,j) &= 0; V_l^n(D,j) = 0 \\
V_g^n(M,j) &= \sum_K \Bigg(\frac{y_{K,c}W^{n-1}(K,c) + y_{K,g}W^{n-1}(K,g) + y_{K,p}W^{n-1}(K,p)}{n_l} \\
&\quad + \frac{y_{K,m}W^{n-1}(K,m)W^{n-1}(M,j) + y_{K,pm}W^{n-1}(K,pm)W^{n-1}(M,j)}{n_l} \Bigg) \\
V_l^n(M,j) &= 0 \\
V_g^n(O,j) &= 0 \\
V_l^n(O,j) &= \sum_K \Bigg(\frac{y_{K,c}W^{n-1}(K,c) + y_{K,g}W^{n-1}(K,g) + y_{K,p}W^{n-1}(K,p)}{n_l} \\
&\quad + \frac{y_{K,m}W^{n-1}(K,m)W^{n-1}(M,j) + y_{K,pm}W^{n-1}(K,pm)W^{n-1}(M,j)}{n_l} \Bigg)
\end{aligned} \quad [4]$$

r in the $V(LG,j)$ functions is the ratio that LG players give to each PGG pool. For instance, if $r = 0.25$ then players of strategy LG give a quarter of their endowments to the global PGG and three quarters to the local PGG.

And now to define $W^n(i,j)$. We define this recursively where everyone begins in good standing, or $W^1(i,j) = 1$.

$$W^n(i,j) = W^{n-1}(i,j)\left(1 - lost^n(i,j)\right) + \left(1 - W^{n-1}(i,j)\right) gained^n(i,j) \quad [5]$$

And this can be solved at equilibrium by setting $W^n(i,j) = W^{n-1}(i,j)$:

$$W(i,j) = W(i,j)\,(1 - lost(i,j)) + (1 - W(i,j))\,gained(i,j)$$
$$W(i,j)\,(1 - (1 - lost(i,j)) + gained(i,j)) = gained(i,j)$$
$$W(i,j) = \frac{gained(i,j)}{gained(i,j) + lost(i,j)} \qquad [6]$$

Here $lost^n(i,j)$ and $gained^n(i,j)$ represent the proportion of players using strategy (i,j) who had a good reputation and lost it in round n or had a bad reputation and gained a good one in round n, respectively. Without the superscript n, $gained(i,j)$ and $lost(i,j)$ represent the equilibrium gain and loss of reputation. Note that this is dependent on which of the leading eight strategies of indirect reciprocity which we choose to employ. Each of the leading eight strategies can be calculated the proportion of good players someone aids and defects as well as the proportion of bad players someone aids and defects.

$$A_g(i,j) = X(i,j)\,(1-e) + Y(i,j)e$$
$$D_g(i,j) = X(i,j)e + Y(i,j)\,(1-e)$$
$$A_b(i,j) = (Z(i,j) - X(i,j))\,(1-e) + (1 - Z(i,j) - Y(i,j))\,e$$
$$D_b(i,j) = (Z(i,j) - X(i,j))\,e + (1 - Z(i,j) - Y(i,j))\,(1-e) \qquad [7]$$

Here $X(i,j)$ is the proportion of players which contributed to the MAG and that players with strategy (i,j) will try to give to, $Y(i,j)$ is the proportion of players which contributed to the MAG that players with strategy (i,j) will not try to give to, and $Z(i,j)$ equals all players that players with strategy (i,j) will try to give to, regardless of MAG reputation. These can then be defined for each MAG strategy as follows.

For MAG strategy c:

$$X(i,c) = \sum_{K,k} \frac{y_{K,k} W(K,k)}{n_l}$$
$$Y(i,c) = 0$$
$$Z(i,c) = 1$$

For MAG strategy g:

$$X(i,g) = \sum_{K,k} \frac{y_{K,k} W(K,k) V_g(K,k)}{n_l}$$
$$Y(i,g) = \sum_{K,k} \frac{y_{K,k} W(K,k)\,(1 - V_g(K,k))}{n_l}$$
$$Z(i,g) = \sum_{K,k} \frac{y_{K,k} V_g(K,k)}{n_l}$$

For MAG strategy l:

$$X(i,l) = \sum_{K,k} \frac{y_{K,k} W(K,k) V_l(K,k)}{n_l}$$
$$Y(i,l) = \sum_{K,k} \frac{y_{K,k} W(K,k)\,(1 - V_l(K,k))}{n_l}$$
$$Z(i,l) = \sum_{K,k} \frac{y_{K,k} V_l(K,k)}{n_l}$$

For MAG strategy d:

$$X(i,d) = 0$$
$$Y(i,d) = \sum_{K,k} \frac{y_{K,k} W(K,k)}{n_l}$$
$$Z(i,d) = 0$$

Note that this means $W(i,d) = 0$.

For MAG strategy p:

$$X(i,p) = \sum_{K,k} \frac{y_{K,k} W(K,k) V(K,k)}{n_l}$$
$$Y(i,p) = \sum_{K,k} \frac{y_{K,k} W(K,k) (1 - V(K,k))}{n_l}$$
$$Z(i,p) = \sum_{K,k} \frac{y_{K,k} V(K,k)}{n_l}$$

For MAG strategy m:

$$X(i,m) = \sum_{K,k} \frac{y_{K,k} W(K,k)}{n_l}$$
$$Y(i,m) = 0$$
$$Z(i,m) = \sum_{K,k} \frac{y_{K,k} W(K,k)}{n_l}$$

For MAG strategy pm:

$$X(i,pm) = \sum_{K,k} \frac{y_{K,k} W(K,k) V(K,k)}{n_l}$$
$$Y(i,pm) = 0$$
$$Z(i,pm) = \sum_{K,k} \frac{y_{K,k} W(K,k) V(K,k)}{n_l}$$

These functions are circular and dependent on themselves, with $W(i,j)$ being dependent on $X(i,j)$, $Y(i,j)$ and $Z(i,j)$ and these being dependent on $W(i,j)$. To break this cycle, we assume all $W(i,j)$ in one of these functions is 1, except for MAG defectors in which case it is 0.

Next we can define our functions $gained(i,j)$ and $lost(i,j)$ by different leading eight strategies.

For strategy 1:

$$gained(i,j) = A_g(i,j) + A_b(i,j)$$
$$lost(i,j) = D_g(i,j)$$

For strategy 2:

$$gained(i,j) = A_g(i,j) + A_b(i,j)$$
$$lost(i,j) = D_g(i,j) + A_b(i,j)$$

For strategy 3:

$$gained(i,j) = A_g(i,j) + A_b(i,j) + D_b(i,j)$$
$$lost(i,j) = D_g(i,j)$$

For strategy 4:

$$gained(i,j) = A_g(i,j) + D_b(i,j)$$
$$lost(i,j) = D_g(i,j)$$

For strategy 5:

$$gained(i,j) = A_g(i,j) + A_b(i,j) + D_b(i,j)$$
$$lost(i,j) = D_g(i,j) + A_b(i,j)$$

For strategy 6:

$$gained(i,j) = A_g(i,j) + D_b(i,j)$$
$$lost(i,j) = D_g(i,j) + A_b(i,j)$$

For strategy 7:

$$gained(i,j) = A_g(i,j)$$
$$lost(i,j) = D_g(i,j)$$

For strategy 8:

$$gained(i,j) = A_g(i,j)$$
$$lost(i,j) = D_g(i,j) + A_b(i,j)$$

In returning back to our function of MAG fitness (3), the only remaining undefined functions are $G\left(i,j\right)$ and $H\left(i,j\right)$. Recall $H\left(i,j\right)$ is defined as the proportion of players who someone of strategy $\left(i,j\right)$ aids. This can now be defined easily by using the other functions we have defined above as being:

$$H\left(i,j\right) = A_g\left(i,j\right) + A_b\left(i,j\right) \qquad [8]$$

Defining how much aid a player of strategy $\left(i,j\right)$ receives, or $I\left(i,j\right)$, is somewhat trickier. Here we define it as the sum of aid received from each strategy found in our game:

$$I\left(i,j\right) = \sum_K y_{K,c} + y_{K,g}V_g\left(i,j\right) + y_{K,l}V_l\left(i,j\right) + y_{K,p}V\left(i,j\right) + y_{K,m}W\left(i,j\right) + y_{K,pm}V\left(i,j\right)W\left(i,j\right) \qquad [9]$$

This provides us with all the necessary information to conduct our invasion analysis. The invasion analysis is conducted by comparing resident fitness to invader fitness. These fitnesses are found in equation 1. The PGG component of fitness (2) requires defining PGG behaviour which is itself defined in 4. The MAG component of fitness (3) requires defining MAG reputation, which is built up using the remaining listed functions.

Model Discussion

Because of our invasion analysis, there are only ever two strategies present. This simplifies many of the listed equations, but we chose to write these in their general form above. This also explains why strategies using LG fair poorly in our model. Consider a group where there are only two strategies (G, g) and (LG, g). Looking at equation 9 we see that the amount of aid received by each playstyle can be simplified as $I(G,g) = y_{G,g}V_g\left(G,g\right) + y_{LG,g}V_g\left(G,g\right)$ and $I(LG,g) = y_{G,g}V_g\left(LG,g\right) + y_{LG,g}V_g\left(LG,g\right)$. In comparing these two strategies we notice that the only difference is in $V_g(G,g)$ versus $V_g(LG,g)$, but we know $V_g(G,g) = 1$ and $V_g(LG,g) = r < 1$. So without any more details, we know players using (G,g) will always receive more aid than those using (LG,g). In comparing all strategies a similar pattern emerges where players using LG don't receive as much aid as those using G or L. In reality there are likely to be more than 2 strategies being used at any given time, which allows LG to hedge its bets and potentially out compete those who commit fully to one of the scales. But because of the nature of our analysis that nuance is lost and thus LG is unsustainable.

As for strategies M and O, as we see in the formulas above, these are dependent on having received MAG aid. We find that these strategies are more resistant to defectors because if they stop receiving aid from defectors they will in turn stop subsidizing these defectors. However, when invading or being invaded by pure cooperators they perform worse. The reason for this is the implementation error in providing MAG aid. If we take as an example a player using M, who gives to the global PGG when they've been aided. Even if all players want to aid global cooperators, they will sometimes fail to do so because of the implementation error. In such cases, if the M player doesn't receive aid, then the next round they will purposely defect from the PGG and then again fail to receive MAG aid. Because the implementation error also makes it that sometimes a player gives aid when they didn't mean to, there's a chance that the M who didn't receive aid once and started defecting could later on receive aid and restart cooperating. Otherwise, we may expect all players using a strategy of M and O to eventually defect because of one error. This means that M and O will provide less to the PGG than G and L and will receive less aid in the MAG, and thus under most conditions we find that the more cooperative strategies beat their stricter counterparts. A more lenient form of M and O which only defects if they weren't aid two rounds in a row may avoid this defection trap, but this falls outside the scope of our current model.

Finally, the model results were not greatly changed by considering different leading eight strategies. As can be seen in the equations above, the strategy we used (strategy 1) is more lenient than certain others seeing as there's only one way to lose standing. Strategies such as 8 are especially harsh seeing as there's only one way to gain standing and multiple ways to lose it. In our analysis, we find that the results remain the same for the majority of strategies and only change for intermediate strategies which are themselves out competed.

In particular, if we focus on the standing (L1), consistent standing (L2), and staying (L7) strategies, which are highlighted by Hilbe et al. (2018) as being the most resilient to noisy reputations, then when assigning specific parameters the only difference in the single invader analysis occurs for strategies (D, pm) and (LG, pm) and in the group level analysis for strategy (LG, pm). Given that these strategies are themselves outcompeted by others, for instance in the single invader model (D, d) outcompetes both (D, pm) and (LG, pm), whether these strategies themselves invade or are invaded by others doesn't change the main results of the model. This is true for all strategies which are changed by using different leading eight strategies. Seeing as the stable evolutionary endpoints remain the same accross strategies, we can say that changing which leading eight strategy will not change the results of the model, only how those results are reached

The analysis was conducted in Python using the SymPy symbolic mathematics computer algebra system (CAS) package and the code for running the analysis can be found in the supplementary information. The full results of the analysis are compiled in an excel spreadsheet, also found in the supplementary information.

Note some of the results are re-evaluated using Mathematica because SymPy was unable to tell whether it was greater or less than 0. For example, (G, l) is able to invade (G, c) in a single invader model when $cm \cdot (1 - e) > 0$. Based on our assumptions about our parameters ($cm > 0$ and $0 < e < 1$) we know this invasion will always occur, but SymPy was unable to detect it as such so we make this change in the results.

Table S1. PGG Strategies

PGG strategy	Description
G	Always contribute to Global PGG
L	Always contribute to Local PGG
LG	Always contribute to both Local and Global PGG
D	Defect from PGG
M	Contribute to Global PGG only if you've been aided in the MAG
O	Contribute to Local PGG only if you've been aided in the MAG

Table S2. MAG Strategies

MAG strategy	Description
c	Always aid
g	Aid if partner contributed to Global PGG
l	Aid if partner contributed to Local PGG
d	Always defect
p	Aid those in good reputation based on PGG contributions
m	Aid those in good reputation based on MAG contributions
pm	Aid those in good reputation based on PGG contributions and MAG contributions

Table S3. Model functions

Function	Definition
$F(i,j)$ 1	Fitness of player using strategy (i,j)
$F_p(i,j)$ 2	Fitness derived from PGG of player using strategy (i,j)
$F_m(i,j)$ 3	Fitness derived from MAG of player using strategy (i,j)
$V_g(i,j)$ 4	Proportion of players using strategy (i,j) who contribute to the global PGG
$V_l(i,j)$ 4	Proportion of players using strategy (i,j) who contribute to the local PGG
$V(i,j)$ 4	Proportion of players using strategy (i,j) who contribute to either PGG. Also defined as $V^g(i,j) + V^l(i,j)$
$W(i,j)$ 6	Proportion of players using strategy (i,j) who are in good standing in the MAG
$H(i,j)$ 8	Proportion of local group members who players using strategy (i,j) will provide aid to in the MAG
$I(i,j)$ 9	Proportion of local group members who provide aid in the MAG to players using strategy (i,j)
$gained(i,j)$	Proportion of players using strategy (i,j) who had a bad reputation and gain a good reputation at equilibrium
$lost(i,j)$	Proportion of players using strategy (i,j) who had a good reputation and gain a bad reputation at equilibrium
$A_b(i,j)$ 7	Proportion of bad reputation players who players using strategy (i,j) will aid
$A_g(i,j)$ 7	Proportion of good reputation players who players using strategy (i,j) will aid
$D_b(i,j)$ 7	Proportion of bad reputation players who players using strategy (i,j) will defect from aiding
$D_g(i,j)$ 7	Proportion of good reputation players who players using strategy (i,j) will defect from aiding
$X(i,j)$	Proportion of good reputation players who players using strategy (i,j) will attempt to aid
$Y(i,j)$	Proportion of good reputation players who players using strategy (i,j) will attempt to defect from aiding
$Z(i,j)$	Proportion of players who players using strategy (i,j) will attempt to aid

Table S4. Model parameters

Parameter	Meaning	Domain
$y_{i,j}$	Proportion of local group members using strategy (i, j)	$0 < y_{i,j} < 1$
a	Number of local groups	> 0
n_l	Number of people per local group	> 0
n_g	Number of people per global group	$= a * n_l$
c_p	Cost of contributing to either PGG	> 0
c_m	Cost of aiding in the MAG	> 0
b_l	Returns from local PGG contributions	$> c_p$
b_g	Returns from global PGG contributions	$> c_p$
b_m	Returns from being aided in the MAG	$> c_m$
e	MAG implementation error rate	$0 < e < 1$
r	Ratio of aid provided to the global PGG as a player using PGG strategy LG	$0 < r < 1$

SI Dataset S1 (General-Assumen-Submission.xlsx)

The provided spreadsheet lists the conditions for a successful invasion given a resident strategy and an invader strategy. Each column represents an invader and each row a resident. Entries show the conditions required for the invader (column) to outcompete and invade the resident (row). A value of 1 represents a guaranteed successful invasion. A value of -1 represents a guaranteed failed invasion. A value of 0 represents a guaranteed tie in fitnesses, also leading to a failed invasion.

The spreadsheet is in 3 pages, one for the single invader analysis, one for the multiple invader analysis, and one for the group invader analysis.

The spreadsheet provided uses the first leading eight strategy and has the ratio for LG set to 0.5, meaning LG players give half their endowment to each of the PGGs. It is possible to use the code provided to generate equivalent sheets for other reputation strategies and LG ratios.

cp = PGG cost
cm = MAG cost
bg = global PGG benefit
bl = local PGG benefit
bm = MAG benefit
e = error rate
nl = local population
a = number of local groups
r = rate of invaders

1 = guaranteed invasion
0 = equal fitness (failed invasion)
-1 = failed invasion
else conditions needed for invasion

SI Dataset S2 (Local_global_analysis_v01.py)

Python code used to generate model predictions, using the SymPy package.
